# *BRAF* mutation is not predictive of long-term outcome in papillary thyroid carcinoma

**DOI:** 10.1002/cam4.417

**Published:** 2015-02-25

**Authors:** Lauren E Henke, John D Pfeifer, Changquing Ma, Stephanie M Perkins, Todd DeWees, Samir El-Mofty, Jeffrey F Moley, Brian Nussenbaum, Bruce H Haughey, Thomas J Baranski, Julie K Schwarz, Perry W Grigsby

**Affiliations:** 1 Department of Radiation Oncology, Washington University School of MedicineSt. Louis, Missouri; 2 Department of Pathology, Washington University School of MedicineSt. Louis, Missouri; 3 Section of Endocrine and Oncologic Surgery, Department of General Surgery, Washington University School of MedicineSt. Louis, Missouri; 4 Department of Otolaryngology, Washington University School of MedicineSt. Louis, Missouri; 5 Division of Endocrinology, Diabetes and Metabolism, Department of Internal Medicine, Washington University School of MedicineSaint Louis, Missouri; 6 Division of Nuclear Medicine, Mallinckrodt Institute of Radiology, Washington University School of MedicineSaint Louis, Missouri

**Keywords:** *BRAF* mutation, *BRAF* mutation survival, BRAF V600, papillary thyroid carcinoma, thyroidectomy

## Abstract

The *BRAF* mutation occurs commonly in papillary thyroid carcinoma (PTC). Previous investigations of its utility to predict recurrence-free survival (RFS) and disease-specific survival (DSS) have reported conflicting results and its role remains unclear. The purpose of this retrospective study was to determine the incidence of the *BRAF* mutation and analyze its relationship to clinicopathologic risk factors and long-term outcomes in the largest, single-institution American cohort to date. *BRAF* mutational status was determined in 508 PTC patients using RFLP analysis. The relationships between *BRAF* mutation status, patient and tumor characteristics, RFS, and DSS were analyzed. The *BRAF* mutation was present in 67% of patients. On multivariate analysis, presence of the mutation predicted only for capsular invasion (HR, 1.7; 95% CI, 1.1–2.6), cervical lymph node involvement (HR, 1.7; 95% CI, 1.1–2.7), and classic papillary histology (HR, 1.8; 95% CI 1.1–2.9). There was no significant relationship between the *BRAF* mutation and RFS or DSS, an observation that was consistent across univariate, multivariate, and Kaplan–Meier analyses. This is the most extensive study to date in the United States to demonstrate that *BRAF* mutation is of no predictive value for recurrence or survival in PTC. We found correlations of *BRAF* status and several clinicopathologic characteristics of high-risk disease, but limited evidence that the mutation correlates with more extensive or aggressive disease. This analysis suggests that *BRAF* is minimally prognostic in PTC. However, prevalence of the *BRAF* mutation is 70% in the general population, providing the opportunity for targeted therapy.

## Introduction

Papillary thyroid carcinoma (PTC) is the most common endocrine malignancy 1. The incidence of PTC has increased over the past several decades and now comprises 88% of all thyroid carcinomas 2–5. The course is fairly indolent and treatment is curative, typically involving surgery followed by radioactive Iodine^131^ (RAI) administration 2,6. However, a select patient population displays a more aggressive phenotype marked by disease recurrence or death due to thyroid cancer 2. Efforts have been made to identify patients at risk and to develop methods to distinguish markers, such as somatic mutations, that might predict for good versus poor prognosis.

One such mutation that has been extensively studied is the *BRAF* gene mutation. The majority of these mutations involve a transversion from T to A at nucleotide 1799, leading to a valine to glutamic acid change in codon 600. This results in the *BRAF*^*V600E*^ mutation which is the most common mutation in PTC. It occurs in about 70% of patients 7–9. Additional activating mutations in V600, such as V600K and V600D are well described but account for only a minority of cases 10–12. Their exact incidence in a thyroid cancer-specific setting is not established. Multiple publications have reported associations between *BRAF* V600 mutations (hereafter referred to collectively as *BRAF*) and poor prognosis 13–16. It has been reported that the *BRAF* mutation correlates with increased disease recurrence and disease-specific mortality 9,17–19. However, several recent studies have failed to corroborate these findings, leaving the overall significance of the *BRAF* mutation unclear 20–24.

Much of the literature investigating the role of the *BRAF* mutation in outcomes of PTC has been limited by the shortcomings of meta-analysis or has been otherwise limited by low patient numbers or short follow-up. In a disease such as PTC, where negative events such as recurrence and disease-related mortality are rare and often occur late, a large patient cohort and extended follow-up time are critical to the strength of analysis. As such, while some groups have suggested that PTC patients should be routinely tested for the *BRAF* mutation in order to guide therapy, the prognostic utility of the *BRAF* mutation as a predictor of recurrence or mortality has not been strongly established 25,26.

This retrospective study, with the largest cohort of patients and longest follow-up time of any single institution in the United States to date, analyzed the relationship between the *BRAF* mutation and PTC. Clinical and pathological outcomes, including disease recurrence and disease-specific mortality, and *BRAF* mutational status were analyzed for possible correlations.

## Materials and Methods

### Patient identification and clinicopathologic data collection

This retrospective study was approved by the Human Research Protection Office at our institution, including retrospective chart review (protocol number 201010705) with waiver of consent. Records of 1712 patients with thyroid cancer who were referred to the department at our facility from 1974 to 2009 were queried. The data set was interrogated for patients with thyroid carcinoma of follicular cell origin who met the following criteria: underwent either partial or total thyroidectomy, received follow-up care, had classic papillary or follicular variant histologic subtypes of PTC, and had available tumor specimens. A total of 508 patients met criteria. Thyroid tumor specimens were obtained from an archived bank of formalin-fixed, paraffin-embedded (FFPE) thyroid tissue. Anaplastic and undifferentiated thyroid carcinomas were excluded from the study. Data abstracted from patient records included histological subtype, treatment records, and clinicopathologic outcomes. Review of records indicated that none of these individuals had any history of therapeutic radiation exposure. *BRAF* mutational status was determined after surgical and medical treatments of all patients were concluded and did not affect treatment decisions.

### PTC histological classification

Hematoxylin and eosin stained slides were examined by study pathologists to identify areas with classic characteristics of PTC, including papillary architecture, typical PTC nuclei (enlarged, overlapping, irregular, ground-glass empty nuclei with nuclear grooves), psammoma bodies, and stromal reaction 27. Histologically, the 508 cases were comprised of classical (*n* = 383) and follicular variant (*n* = 131) subtypes of PTC, classified using standard criteria 27–29. Areas of carcinoma were marked on the glass slides to guide collection of tissue cores from the corresponding FFPE tumor blocks of the case.

### DNA extraction and BRAF mutation analysis

Two tissue cores of 1 mm diameter were extracted from the areas of tumor by means of disposable biopsy punches with plungers (Miltex®, York, PA). Samples were incubated in xylene for three minutes at 50°C; xylene aspiration was followed by two washes with 100% ethanol. Subsequently, samples were incubated for 48 h with 0.5 mg/mL proteinase K (Quiagen®, Germantown, MD), with a mid-interval addition of 0.5 mg/mL proteinase K. DNA was extracted from each sample via a commercial kit (Puregene®, Minneapolis, MN) according to the manufacturer's instructions. Following extraction, DNA was stored at 4°C. Polymerase chain reaction (PCR) was utilized to amplify the 215 base pair (bp) *BRAF* exon 15, using previously published primers and Platinum Taq DNA Polymerase (Invitrogen®, Carlsbad, CA), according to the manufacturer's instructions. Primers (Invitrogen®) were: *BRAF* exon 15F (forward): 5′-TCATAATGCTTGCTCTGATAGGA-3′, BRAF exon 15R (reverse): 5′-GGCCAAAAATTTAATCAGTGGA-3′. PCR conditions consisted of 35 cycles with 1 min of denaturation at 94°C, 1 min of annealing at 51°C, and 1 min of extension at 72°C 30. Samples were then subjected to restriction fragment length polymorphism (RFLP) analysis by the enzyme *TspRI* (Invitrogen®), using the buffer conditions recommended by the manufacturer. *TspRI* cuts the wild-type 215 bp amplification product into two fragments of 120 and 95 bp. Oncogenic *BRAF* V600 point mutations disrupt the restriction site, thereby blocking the cleavage of the amplification product. After the restriction digest, the DNA bands were resolved by agarose gel electrophoresis and visualized by ethidium bromide staining.

### Assay validation

Restriction fragment length polymorphism (RFLP) analysis has been used by numerous other laboratories to evaluate *BRAF* exon 15 30–33. We performed several steps to validate the assay for use in our own laboratory. To establish test accuracy, we compared our results with those of direct DNA sequencing by a commercial clinical reference laboratory (GenPath, Elmwood Park, NJ). For 40 cases of PTC diagnosed between 2009 and 2011 (20 cases with and 20 cases without a *BRAF* V600 mutation) there was 100% concordance between our test and the outside laboratory. These 40 test cases did not overlap with the 508 cases presented here, due to the relative paucity of follow-up data for such recent cases. To establish the reproducibility of the assay, all 40 cases in the validation set were tested three times (in a blinded fashion); there was 100% concordance between test runs. To establish the sensitivity of the test, PCR-amplified DNA from known *BRAF* mutation samples (at 100 nmol/ml) was diluted into *BRAF-*negative DNA (also at 100 nmol/mL); dilution ratios were 1:0, 1:1, 1:2, 1:3, 1:5, 1:10, and 1:20 of *BRAF* mutation positive to negative. These dilutions were then subjected to restriction enzyme analysis and gel electrophoresis, as previously described. The mutant allele could be reproducibly identified when present at an allele frequency of at least 10% in the sample.

### Treatment and follow-up

Most patients underwent total thyroidectomy (97%), 3% had a lobectomy or partial resection, and 0.6% of patients had a thyroid biopsy, only. Surgery for metastatic lymph nodes was performed in 57% of patients, consisting of selective nodal dissection in 46% and modified radical neck dissection in 11%. The remaining 43% had no surgical removal of neck lymph nodes. Postoperative ^131^I was administered to 94% of patients. Nine additional patients were treated with ^131^I after recurrence. The administered activity of ^131^I for treatment was based on standard guidelines for adult patients 34,35. Surveillance consisted of physical examination and laboratory studies including thyroid stimulating hormone (TSH), triiodothyronine, and free thyroxine with the addition of thyroglobulin levels in the latter years of the study.

### Statistical analysis

Clinical and pathological outcomes and *BRAF* mutational status were analyzed for significant associations. *P* < 0.05 was considered statistically significant and all *P* values were two-tailed. *T* tests were used for comparison of data with continuous variables, while Chi-squared tests were used for dichotomous data. All variables associated with the *BRAF* mutation, disease recurrence, or disease-specific survival (DSS) at the *P < *0.05 level were entered into multivariate logistic regression models for *BRAF* positivity. To remove redundancy and improve the predictive value of the multivariate analysis, complex variables such as AJCC Stage were reduced to the variables they comprise, such as histologic tumor size, extrathyroidal extension, and location of disease at diagnosis. Kaplan–Meier analysis was also performed to estimate the recurrence-free survival and DSS probabilities for *BRAF* mutation positive versus negative patient groups. Statistical analyses were performed using SAS, Version 9.2 (SAS Institute, Inc., Cary, NC).

## Results

### Patient characteristics

A total of 508 patients meeting eligibility criteria with evaluable tissue underwent *BRAF* gene analysis. Patient and tumor characteristics are illustrated in Table1. Average age at diagnosis was 45.5 ± 15.1 years (range 5.8–84.6 years, median 45.1 years) and mean follow-up time was 9.8 years with a median follow-up time of 8.0 years (range of 0.1–40.1 years). Pathological findings included thyroid capsular invasion in 47%, vascular invasion in 15%, microscopic positive margins in 28%, soft tissue invasion in 30%, and bilateral thyroid involvement in 36%. The average histologic tumor size was 2.0 ± 1.7 cm at the largest dimension. The majority of patients had disease limited to the thyroid only (54.8%, 278/507), 42.2% had disease in the neck nodes and thyroid, 2.8% had distant metastases to the lung, and 0.2% had disease in the thyroid and bone metastases.

**Table 1:** Patient, tumor and treatment characteristics stratified by *BRAF*^*V600*^ mutation

Characteristic	Number (%) or median (range)	BRAF^V600^ positive (%)	BRAF^V600^ negative (%)	*P* value
All patients	508 (100)	340 (66.9)	168 (33.1)	NA
Gender				
Male	125 (24.6)	88 (70.4)	37 (29.6)	0.342
Female	383 (75.4)	252 (65.7)	131 (34.2)	
Age at diagnosis (years)	45.1 (5.8–84.6)	45.5 (5.8–81.6)	45.1 (13.9–84.6)	0.679
Race				
White	423 (83.3)	285 (67.4)	138 (32.6)	0.714
Black	50 (9.8)	33 (66.0)	17 (34.0)	
Asian	25 (4.9)	17 (68.0)	8 (32.0)	
Hispanic	10 (2.0)	5 (50.0)	5 (50.0)	
Histology				
Classic papillary	377 (74.2)	266 (70.6)	111 (29.4)	0.003
Follicular variant	131 (25.8)	74 (56.5)	57 (43.5)	
Pathological features				
Thyroid capsule invasion	240 (47.3)	181 (59.2)	59 (30.5)	0.001
Soft tissue invasion	151 (29.8)	118 (78.2)	33 (21.9)	<0.001
Vascular invasion	74 (14.6)	54 (73.0)	20 (27.0)	0.223
Positive margins	144 (28.4)	110 (76.4)	34 (23.6)	0.004
Tumor size (cm)	1.5 (0.1–13.0)	1.5 (0.1–9.2)	1.5 (0.2–13.0)	0.600
Multifocal	233 (46.0)	153 (65.7)	80 (34.3)	0.597
Cervical LN involvement	228 (45.0)	169 (74.1)	59 (25.9)	0.002
Extent of disease				0.004
Thyroid only	278 (54.8)	170 (61.2)	108 (38.9)	0.002
Thyroid and cervical LN	214 (42.2)	161 (75.2)	53 (24.8)	<0.001
Lung metastases	14 (2.8)	7 (50.0)	7 (50.0)	0.172
Bone metastases	1 (0.2)	1 (100)	0 (0)	0.482
AJCC tumor stage				
T1	217 (39.8)	139 (63.4)	78 (36.6)	0.037
T2	126 (26.3)	77 (58.0)	49 (42.0)	
T3	55 (12.1)	42 (69.6)	13 (30.4)	
T4	106 (21.9)	80 (71.2)	26 (28.8)	
AJCC nodal stage				
N0	279 (57.5)	172 (58.1)	107 (41.9)	0.015
N1a	164 (30.8)	119 (72.2)	45 (27.8)	
N1b	62 (11.7)	48 (76.1)	14 (23.9)	
Initial I-131 dose (mCi)	150 (0–980)	150 (0–980)	150 (0–950)	0.498
Total I-131 dose (mCi)	150 (0–980)	150 (0–980)	150 (0–950)	0.681
Follow-up time (years)	8.0 (0.1–40.1)	8.0 (0.1–30.2)	7.8 (1.1–40.1)	0.306

LN, lymph node; AJCC, American Joint Committee on Cancer.

### BRAF analysis

**Figure 1:**
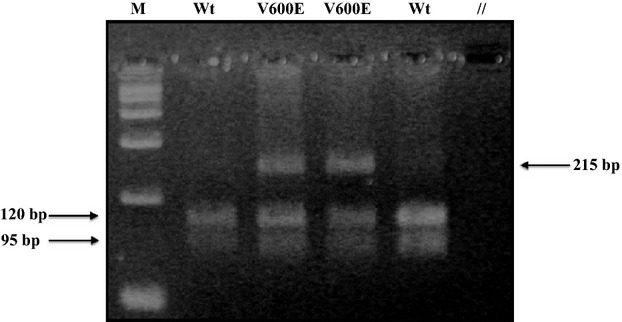
*BRAF*^V600^ evaluation. Representative analysis of FFPE thyroid tissue specimens. DNA from four samples was amplified by PCR and submitted to RFLP by incubation with the restriction enzyme TspRI. Two samples displayed mutated (V600E, V600K, etc.) digestion pattern, with three fragments of 215 bp (undigested, mutated allele), 120 and 95 bp. Two samples displayed the wild-type (Wt) digestion pattern, showing two fragments of 120 and 95 bp, respectively. M, molecular marker; //, empty lane.

The *BRAF* mutation was present in 66.9% of patients (340/508). Mutation status was identified by RFLP, which displayed two, wild-type DNA bands (120 and 95 bp) digested by the *TspRI* restriction enzyme when the *BRAF* V600 mutation was absent, and three DNA bands (215, 120, and 95 bp) when the *BRAF* V600 mutation was present (heterozygote). Representative cases are illustrated in Figure1. Follow-up time did not differ between *BRAF*-positive and *BRAF-*negative patients (*P* = 0.507). Initial RAI dose also did not differ between *BRAF*-positive and -negative patients (*P *=* *0.824). The presence of the *BRAF* mutation was associated with classic papillary histology (*P *=* *0.003), capsular invasion (*P *=* *0.001), soft tissue invasion (*P *<* *0.001), positive margins after surgery (*P *=* *0.004), cervical lymph node involvement (*P* = 0.002), and tumor location at diagnosis (*P* = 0.004) (Table1). On multivariate analysis, only capsular invasion (HR, 1.7; 95% CI, 1.1–2.6), classic papillary histology (HR, 1.8; 95% CI 1.1–2.9), and cervical lymph node involvement (HR, 1.7; 95% CI, 1.1–2.7) at the time of diagnosis were independently predictive of the *BRAF* mutation.

### Patient outcome

**Figure 2:**
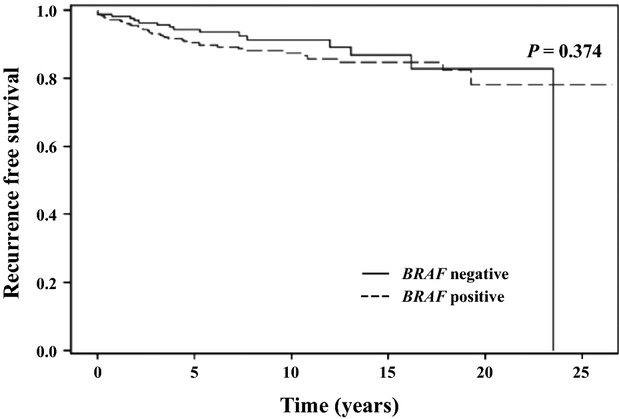
Progression-free survival based on *BRAF* mutational status.

**Figure 3:**
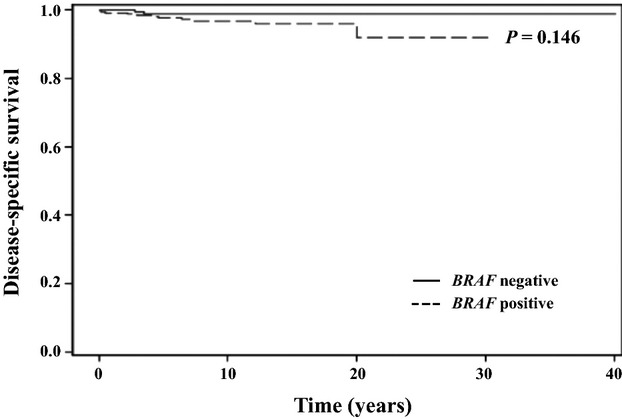
Disease-specific survival based on *BRAF* mutational status.

Overall survival for the entire cohort at 10 and 20 years was 95.4% and 84.5%, respectively. A total of 49 patients died during the study period and 13 of these deaths (27%) were due to thyroid cancer. Disease-specific survival at 10 and 20 years was 97.4% and 96.8%, respectively. Recurrence-free survival (RFS) at 10 and 20 years was 88.8% and 80.3%, respectively. There was no difference in the probability of RFS in patients with the *BRAF* mutation than in patients without the mutation (Fig.2). Similarly, the probability of DSS was not different in patients with or without the *BRAF* mutation (Fig.3). These findings were consistent with the lack of association observed between the *BRAF* mutation and increased risk of recurrence or cancer-related mortality on univariate and multivariate analyses.

Numerous known correlates of high-risk disease were associated with increased risk for recurrence and disease-specific mortality (Table2). For recurrence and cancer-specific death, significantly associated pathological features included capsular invasion, vascular invasion, soft tissue invasion, positive surgical margins, and histologic tumor size (Table2). Tumor involvement of the cervical lymph nodes or lung at diagnosis was also associated with increased recurrence and mortality, whereas tumor confined to the thyroid was negatively correlated with both outcomes (Table2). Male gender was associated with disease recurrence (*P *=* *0.009) only, while increasing age was associated with increased risk of cancer-specific death (*P *<* *0.001).

**Table 2:** Univariate analyses of clinicopathologic features for association with disease recurrence and cause-specific survival

	Risk of recurrence				Risk of death			
	Univariate		Multivariate		Univariate		Multivariate	
Characteristic	Hazard ratio for recurrence (95% CI)	*P* value	Hazard ratio for recurrence (95% CI)	*P* value	Hazard ratio for disease-specific mortality (95% CI)	*P* value	Hazard ratio for disease-specific mortality (95% CI)	*P* value
*BRAF* mutation	1.3 (0.7–2.3)	0.376	Eliminated	NS	2.9 (0.6–13.1)	0.165	Eliminated	NS
Age at diagnosis	1.0 (0.98–1.01)	0.653	Eliminated	NS	1.1 (1.0–1.1)	<0.001	8.2 (1.8–37.3)	<0.001
Multifocal disease	1.5 (0.9–2.6)	0.112	Eliminated	NS	3.4 (0.9–12.6)	0.066	Eliminated	NS
Classic papillary histology	2.7 (1.1–6.2)	0.024	2.6 (1.1–6.4)	0.035	3.5 (0.5–27.0)	0.230	Eliminated	NS
Male gender	2.0 (1.2–3.5)	0.009	Eliminated	NS	2.6 (0.9–7.7)	0.088	Eliminated	NS
Disease location								
Thyroid only	0.2 (0.1–0.4)	<0.001			0.1 (0.01–0.6)	0.013		
Nodal involvement	2.8 (1.6–5.0)	<0.001	Eliminated	NS	2.0 (0.6–6.0)	0.235	Eliminated	NS
Lungs	7.8 (3.3–18.3)	<0.001			22.5 (6.7–75.1)	<0.001		
Bone	0 (0)	0.986			0 (0)	0.993		
Soft tissue invasion	3.5 (2.1–6.0)	<0.001	Eliminated	NS	5.0 (1.5–16.6)	0.009	Eliminated	NS
Positive margins	2.9 (1.8–4.6)	<0.001	Eliminated	NS	7.5 (2.0–27.8)	0.002	8.2 (1.8–37.3)	0.006
Thyroid capsule invasion	4.6 (2.4–8.6)	<0.001	2.2 (1.1–4.4)	0.028	13.0 (1.7–100.7)	0.014	Eliminated	NS
Vascular invasion	3.6 (2.0–6.2)	<0.001	2.1 (1.2–3.8)	0.01	3.8 (1.2–12.0)	0.023	3.7 (1.05–13.6)	0.042
Cervical LN involvement	4.6 (2.5–8.8)	<0.001	2.8 (1.4–5.4)	0.004	13.7 (1.8–105.6)	0.012	Eliminated	NS
Histologic tumor size	1.3 (1.2–1.4)	<0.001	1.2 (1.2–1.5)	<0.001	1.4 (1.2–1.6)	<0.001	Eliminated	NS

LN, lymph node.

On multivariate analysis, only histologic tumor size (HR, 1.3; 95% CI 1.2–1.5), capsular invasion (HR, 2.2; 95% CI 1.1–4.4), vascular invasion (HR, 2.1; 95% CI 1.2–3.8), classic papillary histology (HR, 2.6; 95% CI 1.1–6.4), and cervical lymph node involvement (HR, 2.8; 95% CI 1.4–5.4) remained independent risk factors for recurrence. Independent predictors of disease-specific mortality included vascular invasion (HR, 3.8; 95% CI 1.05–13.55), positive surgical margins (HR, 8.2; 95% CI 1.8–37.3), and increased age at diagnosis (HR, 1.1; 95% CI 1.1–1.2).

## Discussion

This study sought to clarify the relationship between the *BRAF* mutation and long-term outcome in PTC. Previously, the *BRAF* mutation has been reported to indicate a poorer prognosis in PTC although there have been significant inconsistencies between various studies 17–24. In our analysis, we found a consistent lack of relationship between the *BRAF* mutation and either disease recurrence or disease-specific mortality. This was uniformly observed across univariate, multivariate, and Kaplan–Meier analyses.

The method we used to test for the V600E mutation was restriction fragment polymorphism analysis (RFLP). It was the method used by some large reference labs until early 2009 and thus has demonstrated clinical accuracy. It is noteworthy that during most of the time frame covered by the studies in our paper discussing correlations in outcome with BRAF mutations had a physician outside of an academic medical center ordered mutational analysis, it would likely have been done by RFLP analysis. Testing has shifted from RFLP to direct DNA sequence analysis in large reference laboratories because a test kit that has FDA approval has become available, not because of accuracy concerns with RFLP.

A majority of the patients in our cohort (67%) had the *BRAF* mutation, which is consistent with populations represented in the literature 7–9. It has been suggested that this high prevalence of the *BRAF* mutation in PTC suggests an important role for the mutation in PTC-related morbidity and mortality 18. However, we propose the opposite. Given the rarity of recurrence and disease-specific mortality in PTC (10% and 5% of patients, respectively) and the contrastingly high prevalence of the mutation (70%, on average, in the literature), an absence of a link between the *BRAF* mutation and these negative events seems logical 2,7–9. Importantly, the lack of prognostic value of the *BRAF* mutation observed in this study is not attributable to difference in follow-up time or treatment between *BRAF*-positive and -negative patient groups (Table1).

We also evaluated the association between known clinicopathologic factors of poor prognosis and the *BRAF* mutation. While multiple poor prognostic factors correlated with *BRAF* mutations on univariate analysis (Table1), only capsular invasion, cervical lymph node involvement, and classic papillary histology remained independently predictive of the *BRAF* mutation on multivariate analysis 13–16. All three have previously been described to predict for *BRAF* mutations 13–16,19. The significant association between the *BRAF* mutation and the classic papillary histologic sub-type of PTC (the most common sub-type) in this analysis, in comparison to the follicular variant sub-type, is a well-documented relationship 13,20,21,24,26,36.

Compared to studies that used univariate analysis, our multivariate analysis revealed few risk factors that correlated with the *BRAF* mutation. A recent publication detailing the University of California, San Francisco experience has called into question the relationship between the *BRAF* mutation and poor clinicopathologic factors. In the analysis of a cohort of 429 patients, Gouveia, et al. found a minimal association between the mutation and negative prognostic indicators on multivariate analysis 24,37.

In addition, we analyzed the associations between common clinicopathologic features, RFS, and DSS. The strongest predictor of increased risk on multivariate analysis for recurrence was cervical lymph node involvement, followed by classic papillary histology, capsular invasion, vascular invasion, and then histologic tumor size. These are well-documented risk factors and are components of the current AJCC staging system 9,13,19,24,34,35. Our results suggest that the current means of initial prognostication for PTC already accounts for the greatest established predictors of recurrence. For cancer-specific death, the strongest predictors were older age at diagnosis, positive surgical margins, and vascular invasion 9,17–19,22. Advanced age and positive margins were associated with an eightfold increase of disease-specific mortality.

The greatest strengths of this analysis are its extensive follow-up time (mean 9.8 years, median 8.0 years, range 40.1 years) and large cohort. The bulk of recurrences and disease-specific deaths in our cohort occurred at least 2.5 years (and up to 23.5 years) after diagnosis. These events were also rare, occurring in only 11.0% and 2.6% of patients, respectively. If the range of follow-up time of our study had been limited to 67 months, the follow-up range in one of the most-cited meta-analyses, we would have failed to capture 25% of disease recurrences and 54% of the cancer-specific deaths in our cohort 19. Thus, the importance of having a large cohort and extensive follow-up data in this study cannot be emphasized strongly enough. One weakness of this analysis is its retrospective nature. However, all patients were treated homogeneously, following guidelines at a single, academic institution.

Our data confirm the high incidence of the *BRAF* mutation in our population. Approximately 70% of patients will have the mutation. Although the presence of the mutation is not independently predictive of poor outcome, a significant percent of *BRAF-*positive patients will develop recurrence and metastatic disease. Therefore, treatments guided by presence of the mutation are warranted. Such therapies might include increased cervical nodal dissection and use of *BRAF* inhibitors. Both in this patient cohort and in previous reports, preoperative *BRAF* mutation status in cytology specimens has correlated with lymph node status, and some suggest it could guide the extent of node removal during surgery 38. We propose that the clear role for *BRAF* testing lies in patients who suffer multiple recurrences or develop radioactive iodine (RAI) resistance. Approximately two-thirds of those patients (those with the *BRAF* mutation) might benefit from targeted therapy with *BRAF* inhibitors, which have been developed for treatment of metastatic melanoma. *BRAF* inhibitors have shown promising results against thyroid cancer cells with *BRAF* mutations in vivo 39. Additionally, preliminary results from the phase III DECISION trial demonstrate that Sorafenib, a non-specific active *BRAF* inhibitor, significantly improves PFS compared to placebo in patients with progressive RAI-refractory differentiated thyroid carcinoma 40. However, the DECISION trial included both *BRAF-*positive and *BRAF-*negative patients, a factor that is likely to significantly reduce the observed efficacy of *BRAF* inhibitors in this population. A randomized trial to evaluate the safety and efficacy of *BRAF* inhibitors to improve progression-free survival in progressive RAI-refractory PTC patients, with stratification of patients into sub-groups on the basis of *BRAF* mutational status is warranted.
